# The Potential to Fight Obesity with Adipogenesis Modulating Compounds

**DOI:** 10.3390/ijms23042299

**Published:** 2022-02-19

**Authors:** Jiaqi Zhao, Ailin Zhou, Wei Qi

**Affiliations:** Gene Editing Center, School of Life Science and Technology, ShanghaiTech University, Shanghai 201210, China; zhaojq@shanghaitech.edu.cn (J.Z.); zhoual@shanghaitech.edu.cn (A.Z.)

**Keywords:** obesity, adipogenesis, AMPK, BMP, epigenetics, sirtuin

## Abstract

Obesity is an increasingly severe public health problem, which brings huge social and economic burdens. Increased body adiposity in obesity is not only tightly associated with type 2 diabetes, but also significantly increases the risks of other chronic diseases including cardiovascular diseases, fatty liver diseases and cancers. Adipogenesis describes the process of the differentiation and maturation of adipocytes, which accumulate in distributed adipose tissue at various sites in the body. The major functions of white adipocytes are to store energy as fat during periods when energy intake exceeds expenditure and to mobilize this stored fuel when energy expenditure exceeds intake. Brown/beige adipocytes contribute to non-shivering thermogenesis upon cold exposure and adrenergic stimulation, and thereby promote energy consumption. The imbalance of energy intake and expenditure causes obesity. Recent interest in epigenetics and signaling pathways has utilized small molecule tools aimed at modifying obesity-specific gene expression. In this review, we discuss compounds with adipogenesis-related signaling pathways and epigenetic modulating properties that have been identified as potential therapeutic agents which cast some light on the future treatment of obesity.

## 1. Introduction

Obesity as a chronic condition has nearly tripled in the global population since 1975. In 2016, 39% of adults globally aged 18 years and older were overweight (BMI ≥ 25 kg/m^2^) and 13% were obese (BMI ≥ 30), according to the World Health Organization. As for children, 39 million under the age of 5 were overweight or obese in 2020. Obesity increases the likelihood of developing obesity-related disorders, such as cardiovascular disease (CVD), type 2 diabetes mellitus (T2DM), nonalcoholic fatty liver disease (NAFLD), asthma, and others. Obesity is also associated with poor socioeconomic status and malnutrition. In the past two years, with the impact of the global COVID-19 pandemic, there is emerging evidence that obesity is also a risk factor for severe COVID-19 complications [1]. All these pieces of evidence demonstrate that obesity is an increasing burden on the healthcare and economic systems.

The fundamental cause of obesity is energy overconsumption and/or inadequate energy expenditure. Excessive food/energy intake leads to the expansion of adipose tissues, including both increased numbers of fat cells, i.e., adipogenesis, and increased size of individual adipocytes (hypertrophy). In mammals, there are at least three classes of adipocytes: white, beige, and brown adipocytes [2,3]. White adipose tissue (WAT) depots are broadly distributed intra-abdominally or subcutaneously, and composed of white adipocytes characterized by a large unilocular lipid droplet for energy storage in the form of triglycerides, which is their primary function. Proper systemic stimuli of starvation or lack of energy triggers lipolysis and energy release from white adipocytes as free fatty acids. White adipocytes also secrete adipokines, such as adiponectin and leptin, to modulate organismal energy homeostasis.

In contrast, brown and beige adipocytes exhibit multilocular intracellular lipid droplets and are rich in mitochondria. The most commonly used molecular marker for brown and beige adipocytes since the earliest studies is the high expression level of uncoupling protein 1 (UCP1), which is a proton transporter/channel localized in the inner membrane of the mitochondria, and involved in stimulated heat production in response to cold and other difficult conditions through proton leak [2,3]. Thus, they are also called thermogenic adipocytes. In mice, beige adipocytes usually exist sporadically in subcutaneous WAT depots, while typical brown fat is found in the interscapular depot and developmentally originates from Myf5+ precursors. Recent advances in rodent lineage-tracing in vivo studies have suggested that the various adipose tissue depots along the body are developed embryonically from discrete origins, which was recently well summarized [4]. In adult humans, thermogenic adipose tissues usually reside in the neck, supraclavicular, and para-vertebrate areas, which can be visualized using a fluorine-18 fludeoxy-glucose (^18^F-FDG) label and positron emission tomography (PET) imaging, although the exact nature of brown or beige adipocytes is unclear.

Due to the energy-consuming feature of thermogenic adipocytes, it has been proposed that promoting brown/beige adipogenesis would be a therapeutic strategy to treat obesity, which is under intensive investigation. On the other hand, as white adipocytes mainly store energy and constitute the biggest pool of gained fat in obesity, an increased number of white adipocytes in obesity is considered a metabolically healthy form compared with that of adipocyte hypertrophy. Larger adipocytes may experience mechanical and hypoxia stress and exhibit higher lipolysis and inflammatory-cytokine secretion [5]. Therefore, modulating excessive white adipogenesis may also be beneficial.

As studies of the anatomical, mechanistic, and functional aspects of adipose tissues have provided a relatively comprehensive map of adipogenesis and comprehensive details of the mechanisms, researchers have been trying to discover natural or synthetic small molecular weight (SMW) compounds or biologic molecules to treat obesity or related metabolic diseases through modulating adipogenesis. On the other hand, there are natural food supplements or repurposed drugs that may stimulate brown/beige adipogenesis and exhibit anti-obesity activity. In fact, there are various mechanisms/pathways affecting fat accumulation in obesity, such as signaling pathways, chronic inflammation, insulin resistance, mitochondrial dysfunction, circadian rhythm disturbance, and so on. Here, we briefly outline the cellular and molecular mechanism of adipogenesis and discuss some of the signaling and epigenetic compounds modulating the adipogenesis process, as this may lead to potential therapeutic methods in the treatment of obesity in the future. In addition, research using these as tools provides critical information on the targetable signaling pathways or epigenetic regulators needed to treat obesity, even though some may function systemically in multiple facets.

## 2. Mechanism of Adipogenesis

**Figure 1:**
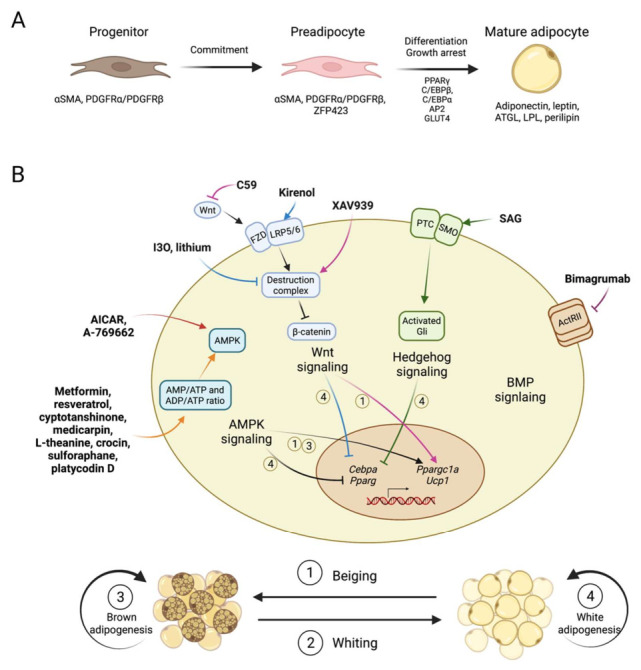
Compounds regulating adipogenesis through various signaling pathways. (**A**). Overview of the process of adipogenesis. Progenitor marked by αSMA, PDGFRα/PDGFRβ will firstly make a commitment to preadipocyte, and then differentiate into mature adipocyte. During the differentiation process, some transcription factors that are critical to adipogenesis, like PPARγ, C/EBPβ, C/EBPα et al., are required. (**B**). Compounds regulating adipogenesis through various signaling pathway. Adipogenesis-regulating compounds are shown in bold, and their pathway-modulating mechanisms are presented briefly. For regulated adipogenesis, the four processes including beiging, whiting, brown adipogenesis and white adipogenesis are of concern. Beiging stands for elevated thermogenic ability and increased brown adipocyte characteristics in white adipocyte or white adipose tissue. Whiting stands for the loss of thermogenic ability and brown adipocyte characteristics of brown adipocyte or brown adipose tissue. Brown adipogenesis stands for increased brown adipocyte differentiation or thermogenic ability in brown adipocytes or brown adipose tissue. White adipogenesis stands for increased white adipocyte differentiation in white adipocytes or white adipose tissue. In general, the activation of the AMPK signaling pathway through certain compounds is shown to inhibit white adipogenesis, and promote beiging of white adipogenesis and brown adipogenesis. Activation of the canonical Wnt signaling pathway through certain compounds is shown to inhibit white adipogenesis, and its inhibition is shown to promote beiging of WAT. Activation of the Hedgehog signaling pathway is shown to inhibit white adipogenesis.

Adipogenesis describes the process of the differentiation of adipocytes, in which a variety of factors play important roles. Research over more than three decades has revealed that adipogenesis is a complex process, with primarily two steps (Figure 1A). The first step is the commitment to preadipocytes from the mesenchymal stem cells (MSCs), which usually reside in the vesicular structure of adipose tissue and are positive for the molecular markers’ platelet-derived growth factor receptor (PDGFR) α or/and PDGFRβ [5]. In the network regulating the commitment of MSCs, early studies identified the comprehensive interplay of multiple pathways including MAPK, transforming growth factor-β (TGF-β), AMP-activated protein kinase (AMPK), bone morphogenetic proteins (BMPs), Wnt, and Hedgehog pathways, which have been shown to perform multiple functions, such as stemness program, cell proliferation and metabolic states regulation. For instance, BMP2 and BMP4 promote commitment and drive in vitro adipogenic differentiation from mouse embryonic fibroblast (MEF) [5]. On the contrary, Wnt signaling promotes stemness and inhibits adipogenesis [5]

The second step in adipogenesis is the differentiation from preadipocyte and maturation into a functional adipocyte. At the center of this differentiation regulation network are two principal adipogenic TFs, PPARγ and CEBPα, which drive in a coordinated manner the entire terminal differentiation process and control the transcriptional activation of many markers for mature adipocytes, such as insulin receptor, adiponectin and fatty acid binding protein (FABP4, also referred to as aP2) [6]. These are required for adipogenesis both in vitro and in vivo. Commitment signals also upregulate their expression levels. There are a few other transcription factors promoting adipogenesis in the network, including CEBPβ, RXRα/β, and STAT5. CEBPβ may compensate for the loss of CEBPα in embryonic adipogenesis, but not in adult adipogenesis [7]. As a typical nuclear receptor, PPARγ forms heterodimer with RXR for DNA binding and transcriptional regulation [6]. Working together with these TFs, multiple layers of epigenetic regulators, such as DNA demethylases, histone modifiers and multiple microRNAs, participate in the adipogenesis process. Their roles can be facilitating or restricting to the adipogenic program and are detailed later.

In addition to the common factors for adipogenesis, the differentiation of brown/beige adipocytes is under the modulation of unique TFs and epigenetic regulators. EBF2 is one of the critical brown/beige adipocyte TFs recruiting PPARγ to thermogenic genes [8]. It has also been shown that PR/SET Domain 16 (PRDM16) and Peroxisome proliferator-activated receptor-γ coactivator-1α (PGC1a) determine the fate of brown/beige adipocytes. The more detailed transcriptional mechanisms of adipogenesis have also been elegantly summarized recently elsewhere [2,9].

It is notable that mechanistic understanding in adipogenesis was initially revealed using in vitro cultured cell lines, such as mouse preadipocyte line 3T3-L1 [10] or mouse MSC lines C3H10T1/2 and OP9 [6]. These provide easy-to-handle study materials, make it possible to perform medium- to large-scale compound and genetic screenings, and are widely used in different laboratories. However, they possess innate limitations as they cannot fully capture the adipogenesis in vivo with no external cues or micro-environment in adipose tissue, especially in obesity. Adipose-specific knock-out mouse models using Adipoq-Cre or Fabp4-Cre can be used to validate the importance of the molecular players in adipogenesis and the obesity condition can be mimicked with high-fat diet (HFD) feeding, although individual mouse lines may have limitations [6]. Meanwhile, pharmacological animal studies with SMW chemicals or biological molecules provide another layer of validation, which rely on the specificity and features of the compounds. Most of the studies we review here are carried out in cell culture and animal models. For each molecule, we start by inspecting its functions in adipogenesis-related molecular and cellular mechanism, then extend this to in vivo validation in mice, though only a few compounds entered clinical trials. The gap between animal and human studies also represents one of the major challenges for anti-obesity drug discovery [11].

## 3. Compounds Modulating Adipogenic Signaling Pathways

Adipogenesis in adipose tissues is modulated by many factors, such as signaling pathways, intra-tissue or systemic inflammatory cytokines and immune cells, cold exposure and sympathetic nervous stimulations [12]. Certain adipose depots are more suspectable, for example, interscapular BAT and inguinal WAT are activated by cold exposure. Below, we focus on a few common signaling pathways. Modulating these pathways through chemical molecules or antibodies is showing great potential in promoting brown adipogenesis, enhancing beiging phenotype (beige adipocyte increase/activation in subcutaneous WAT) and improving whole body metabolism (Figure 1B, Table 1).

### 3.1. AMPK Activators

AMPK is an evolutionarily conserved serine threonine kinase composed of a catalytic α-subunit and two regulatory subunits, β and γ. For each subunit, multiple isoforms exist, allowing for up to 12 possible combinations of αβγ complexes in vertebrates. Isoform-specific roles in certain cell types or at specific sub-cellular locations have been reported [13]. AMPK is activated by phosphorylation of Thr172 on α-subunit by upstream kinases, such as LKB1 (STK11) [14] and Calcium/Calmodulin Dependent Protein Kinase 2 (CAMKK2) [15], which can be regulated by β and γ subunits. AMPK can be activated in response to cellular energy level, especially the AMP/ATP and ADP/ATP ratios, which makes it an important energy sensor and the central energy regulation protein at both cellular and body level [16,17,18]. Activated AMPK could inhibit anabolic metabolism pathways and stimulate catabolic pathways, such as by inhibiting lipid and sterol synthesis through phosphorylation of acetyl-CoA carboxylase (ACC) and increasing glucose utilization through phosphorylating proteins in trafficking of glucose transporters [13,19,20]. Importantly, AMPK has also been found to regulate adipogenesis.

Among all AMPK activators, 5-aminoimidazole-4-carboxamide ribonucleoside (AICAR) is a frequently used AMPK direct activator, which works by mimicking AMP and targeting the AMPKγ-subunit [21]. It has been shown that AICAR could inhibit the in vitro differentiation of 3T3-L1, down-regulate adipogenic markers C/EBPα and PPARγ, and inhibit early clonal expansion of pre-adipocytes through activation of AMPK [22]. In addition, 3T3-L1 is an embryonic fibroblast-like preadipocyte, which can be chemically induced to differentiate into mature adipocytes [10]. Further study verified that AICAR administration in vivo led to reduced adipose tissue content and improved glucose tolerance and insulin sensitivity in a diet-induced obesity (DIO) mice model, likely through elevating PGC1α expression in adipose tissue and promoting brown/beige adipogenesis [23]. Later, Vila-Bedmar et al. discovered that activation of AMPK using AICAR promoted brown/beige adipogenesis directly in a murine brown preadipocyte cell line in vitro and in the subcutaneous WAT in mice. Therefore, beiging through AMPK activation could be beneficial in treating obesity or related metabolic diseases [24]. Besides AICAR, A-769662 is another AMPK direct activator targeting the AMPKβ-subunit [25]. Similarly, A-769662 significantly inhibited 3T3-L1 differentiation in vitro [26], while results from an in vivo study showed that A-769662 promoted the browning of inguinal WAT in HFD-fed mice, reduced body weight gain and improved glucose tolerance [27].

Metformin is famous for its multifarious medical employment and has been used in the first-line clinical management of T2D for years. Yang et al. showed that metformin treatment significantly rescued impaired brown adipogenesis through AMPK activation and DNA demethylation on *Prdm16* promoter in neonatal mice from obese mothers, even after excluding metformin’s systemic effects on whole body metabolism [28]. Metformin treatment has been reported to reduce body weight in obese patients without obesity-related diseases [29]. In addition, metformin decreases the T2D risk in obese adults [30], reduces weight gain and the risk of all-cause mortality in overweight patients with T2D [31], and reduces cardiovascular mortality in patients with coronary artery disease [32]. Although AMPK may not be the only target of metformin, the contributions of AMPK activation in these beneficial effects are acknowledged [33].

As AMPK lies on the energy sensing hub, increasing the AMP–ATP ratio would activate AMPK indirectly. There are many natural products that are also AMPK indirect activators, including resveratrol [34], cyptotanshinone [35,36], medicarpin [37], L-theanine [38], crocin [39], sulforaphane [40,41] and platycodin D [42,43] (Table 1). These have all been confirmed to promote brown and beige adipogenesis and/or inhibit white adipogenesis dependent on AMPK activation. Imran et al. showed that cyptotanshinone has a role in up-regulating UCP1, PRDM16 and PGC-1α and promoting brown adipogenesis in both C3H10T1/2 and 3T3-L1 cells by AMPK activation [35]. C3H10T1/2 is another well-established MSC cell line for adipogenic differentiation studies [44]. L-Theanine, a nonprotein amino acid enriched in green tea, was recently found not only to significantly induce the beiging of subcutaneous WAT in mice, but also ameliorate obesity and improve glucose tolerance and insulin sensitivity in HFD-fed mice [38]. Methyl cinnamate, an active component of Zanthoxylum armatum, inhibited differentiation of 3T3-L1 preadipocytes partially through activation of the CaMKK2-AMPK signaling pathway [45].

Other than its effect on adipogenesis, AMPK plays critical roles in multiple metabolic organs including muscle and liver. Systemic activation of AMPK may not be ideal as it may cause the heart muscle to hypertrophy, so alternative approaches are required [13]

### 3.2. Wnt Signaling Modulators

Wnts are a family of cysteine-rich glycoproteins that act as paracrine and autocrine factors to regulate cell growth and cell fate. There are 19 Wnt genes in mammals and most have distinct phenotypes when eliminated from the genome, and such distinct functions of individual Wnt genes may be attributed to their discrete and unique expression pattern [46]. Different Wnts behave in a very similar way when it comes to biochemical signaling mechanisms, which are roughly classified as either canonical (β-catenin-dependent) or non-canonical (β-catenin-independent) pathways. In the canonical Wnt signaling pathway, Wnt ligands bind to a receptor complex of FZD and LRP5/6, which signal through disheveled (DVL) protein to inhibit the kinase activity of “destruction complex” containing glycogen synthase kinase 3β (GSK3β), casein kinase 1α (CK1α), the scaffold protein AXIN and the tumor suppressor adenomatous polyposis coli (APC) to prevent degradation of β-catenin [47]. As a result, accumulated β-catenin trans-locates into the nucleus, serving as a transcription factor that induces activation of T cell factor (TCF)- and lymphoid enhancer factor (LEF)-dependent gene expression [48]. Since Wnt pathways have been shown to play an important role in various diseases, including multiple malignancies [49], neurological diseases [50], inflammatory [51] and fibrotic diseases [52], various compounds have been developed to target Wnt pathways for therapeutic purposes [53].

As early as 2000, Ross et al. found that canonical Wnt signaling plays an important role in adipogenesis [47]. The Wnt signal maintains preadipocytes in an undifferentiated state through inhibition of the adipogenic transcription factors CEBPs and PPARγ in 3T3-L1 cells. They firstly used Wnt-1 and lithium as Wnt pathway activator in vitro, and later demonstrated that the 3T3-L1 cell could express Wnt-10b to resist their own adipogenic program. Later, indirubin-3′-oxime (I3O) was used as a Wnt/β-catenin activator, which works by the same mechanism as lithium in inhibiting GSK3 in the “destruction complex” for β-catenin [46,54,55]. Choi et al. showed that I3O could not only inhibit the differentiation of 3T3-L1, but also inhibit obesity development in DIO mice. Obesity-associated metabolic disorders including hyperlipidemia and hyperglycemia are both ameliorated by I3O treatment with no obvious toxicity [55]. Wnt/β-catenin pathway activator kirenol and 13m also inhibit adipogenesis in 3T3-L1 cells, but their in vivo effect was not verified [56,57].

However, the anti-adipogenesis function of Wnt signaling is the same in brown adipogenesis. Activation of canonical Wnt signaling blocked brown adipogenesis by impairing C/EBPα, PPARγ, FABP4 and UCP1 expression in Rb-/- MEFs and HIB-1B cells, the established brown preadipocyte line [58]. Furthermore, activating Wnt signaling in mature brown adipocytes of mice could stimulates their conversion to white adipocytes, while inhibiting Wnt by C59 or XAV939 in stromal vascular fraction (SVF) from mouse inguinal WAT enhanced thermogenic markers [58,59]. In addition, Wnt signaling is extensively involved in adult stem cell maintenance in the small intestine and other organs. Thus, the Wnt pathway may not be an easy target for obesity treatment.

### 3.3. BMP Pathway Blockers

BMPs belong to the TGF-β family, which is a large family of ligand proteins including TGF-β, nodal, activin, BMPs and growth and differentiation factors (GDFs) playing diverse and important roles in different aspects of development, physiological and pathological conditions [60]. Signaling induced by ligands of this family usually transduce through membrane localized receptor tyrosine kinases (TGF or BMP receptors) and transcription factor Smad proteins, which become phosphorylated, dimerized with Smad4, and translocate to the nucleus in order to modulate target gene transcription. These pathways have long been reported to be related to adipogenesis. For example, Activin A promoted the proliferation of adipocyte progenitors in human multipotent adipose-derived stem (hMADS) cells [61]. Different types of BMPs function in adipogenesis differently, with the most commonly studied BMP4 triggering adipogenic commitment in C3H10T1/2 cells and BMP7 promoting brown/beige adipogenesis in immortalized brown preadipocytes of mice [62,63].

Recent studies show that activin related pathways may have prominent pharmaceutical potential. Bimagrumab, a human blockade antibody inhibitor of activin type II receptors (ActRII) [64], which are receptors for TGF-β family ligands including activins, myostatin and GDF11, is now in clinical trials for obesity treatment. In a phase 2 randomized clinical trial, bimagrumab treatment in overweight patients with T2D led to a significant loss of total body fat mass and to metabolic improvement [65]. As for the mechanism, since the ActRIIB pathway has been shown to act as an important negative regulator in both muscle growth and brown adipocyte differentiation [66,67], ActRIIB blockade by genetic method or Bimagrumab could not only increase muscle size [64], but also specifically increase the amount of BAT and activate its thermogenesis by enhancing mitochondrial oxidative metabolism in mice [66]. Another potential mechanism is, as mentioned above [61], that preventing the interaction between activin A and ActRII could block its downstream signaling, thus controlling the number of undifferentiated adipocyte progenitors and reduce fat mass. The clinical efficacy of Bimagrumab deserves further investigation to understand how Bimagrumab leads to fat loss and metabolic improvement in humans. These insights would provide potential novel targets or mechanisms for future anti-obesity drug discovery.

### 3.4. Hedgehog Pathways

Hedgehog signaling is a highly evolutionary conserved pathway first identified in drosophila [68], playing an important role in animal development. In mammals, at least three types of Hh ligands exist, sonic hedgehog (SHH), Indian hedgehog (IHH) and desert hedgehog (DHH). Binding of Hh ligands to Patched, the Hh receptor, relieves its inhibition on Smoothened (Smo), which is a member of the G protein-coupled receptor (GPCR) superfamily. Such de-repression led to activation by the Gli family of transcription factors [69,70].

Similar to the Wnt pathway, Hedgehog activation blocks both white and brown adipocyte differentiation both in vitro and in mice [71,72]. Besides being an inhibitor of adipogenesis solely, activation of Hh signaling could redirect cell fate, from adipogenic to osteogenic, in mice [73,74]. Since an increase in adipose tissue volume and a decrease in trabecular bone volume in bone marrow has been observed with aging and other osteogenic disorders [75], Hedgehog signaling activators might steer the balance back to bone formation in these conditions. Activation of hedgehog signaling by induced expression of constitutively active Smoothened (SmoM2) or Gli2 (ΔNGli2) in the adipocyte lineage of postnatal mice could prevent obesity induced by a high-fat diet, by suppressing WAT and BAT accumulation [70]. These findings confirmed the important function of Hedgehog signaling in modulating adipogenesis. However, the feasibility of using hedgehog agonist to treat obesity remains to be verified.

### 3.5. Insulin and Other Pathways

Insulin and insulin-like-growth factors (IGFs) share a similar signaling cascade in cells. While targeting different kinds of cell surface receptor tyrosine kinases, both of them transduce signals mainly through insulin receptor substrates 1 and 2 (IRS-1 and IRS-2). Early studies identified insulin and its signaling through IRS-1/2, and the Akt-mTORC1 Ser/Thr kinase cascade was required for adipocyte differentiation of MEFs and 3T3-L1 [76,77,78]. Indeed, insulin is the essential component in the cocktail used for induction and maintenance in in vitro adipocyte differentiation. For validation in vivo, Boucher et al. demonstrated that disrupting insulin and IGF-1 signaling by knocking out their receptors led to impaired adipose tissue development in mice, with white and brown fat mass both decreased. Interestingly, this disruption could endow mice complete resistance to HFD-induced obesity and HFD- and age- induced glucose intolerance, with energy expenditure and basal metabolic rate increased. However, the thermogenic ability of mice brown fat has significant defects [79].

Beside the pathways mentioned above, there are other pathways which are involved in the regulation of adipogenesis and obesity, including fibroblast growth factor (FGF) signaling pathways. FGF has also been examined in early studies for its adipogenesis regulating functions. FGF10 and FGF1 were found to promote the development of white adipose tissue [80,81,82,83]. Furthermore, two FGFR-specific inhibitors, PD-173074 and SU-5402, significantly reduced the differentiation of human preadipocytes [83]. Along this path, further studies are warranted to further validate the modulatory role of these pathways and the mechanisms in vivo.

**Table 1:** Structure, pathway involved, experimental conditions and obesity-related clinical trials of compounds targeting signaling pathways with adipogenesis modulating functions.

Compound	Structure	Pathways Involved	Experimental Conditions	Clinical Trials
AICAR	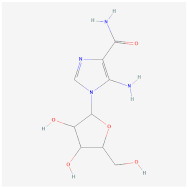	AMPK signaling pathway	**In vitro:** 1. Inhibit the differentiation of 3T3-L1 preadipocyte cell line [22,23]. 2. Promote brown adipogenesis in murine brown preadipocyte cell line [24]. **In vivo:** 1. Promote brown adipogenesis in murine WAT [24]. 2. Reduce adipose tissue content, improve glucose tolerance and insulin sensitivity in diet-induced DIO mice [23].	NCT02322073, registered
A-769662	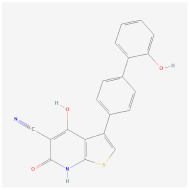	AMPK signaling pathway	**In vitro:** 1. Inhibit the differentiation of 3T3-L1 preadipocyte cell line [26]. **In vivo:** 1. Promote brown adipogenesis in inguinal WAT, reduce body weight gain, improve glucose tolerance in HFD-fed mice [27].	
Metformin	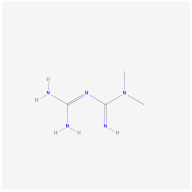	AMPK signaling pathway	**In vivo:** 1. Rescue the impaired brown adipogenesis in neonatal mice from obese mother through AMPK activation [28].	NCT02274948, completed
Cyptotanshinone (from plant *Salvia miltiorrhiza*)	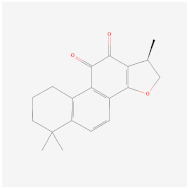	AMPK signaling pathway	**In vitro:** 1. Promote brown adipogenesis in C3H10T1/2 and 3T3-L1 cell line [35]. **In vivo:** 1. Reduce fat accumulation in ob/ob mice [36].	
Medicarpin	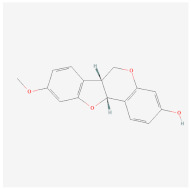	AMPK signaling pathway	**In vitro:** 1. Promote brown adipogenesis in C3H10T1/2 mesenchymal stem cell [37].	
L-Theanine (enriched in green tea)	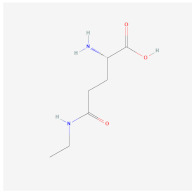	AMPK signaling pathway	**In vitro:** 1. Promote brown adipogenesis of subcutaneous WAT in mice [38]. **In vivo:** 1. Improve glucose tolerance and insulin sensitivity, ameliorate obesity in HFD-fed mice [38].	
Platycodin D (from plant *Platycodon grandiflorum*)	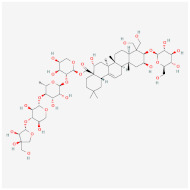	AMPK signaling pathway	**In vitro:** 1. Inhibit the differentiation of 3T3-L1 preadipocyte cell line [43]. **In vivo:** 1. Decrease adipogenic factors in WAT and promote thermogenic factors in BAT of db/db mice [42]. 2. Reduce body weight gain and white adipose tissue weight in db/db mice [42]. 3. Inhibit fat accumulation in HFD-induced obese mice [43].	
Methyl cinnamate (from plant *Zanthoxylum armatum*)	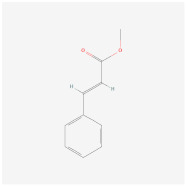	AMPK signaling pathway	**In vitro:** 1. Inhibit the differentiation of 3T3-L1 preadipocyte cell line [45].	
Sulforaphane (enriched in cruciferous vegetables like broccoli and cabbage)	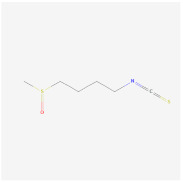	AMPK signaling pathway	**In vitro:** 1. Inhibit the differentiation of 3T3-L1 preadipocyte cell line [40]. **In vivo:** 1. Reduce adipose mass gain and body weight gain in HFD-fed mice [41].	NCT04364360, recruiting
Crocin (from plant saffron and gardenia)	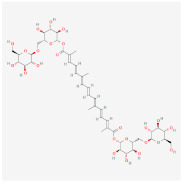	AMPK signaling pathway	**In vitro:** 1. Inhibit the differentiation of 3T3-L1 preadipocyte cell line [39]. **In vivo:** 1. Inhibit adipose formation and reduce fat accumulation in db/db mice [39].	
Lithium		Wnt signaling pathway	**In vitro:** 1. Inhibit the differentiation of 3T3-L1 preadipocyte cell line [47].	
Indirubin-3’-oxime (I3O)	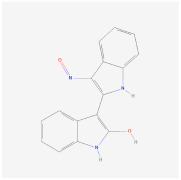	Wnt signaling pathway	**In vitro:** 1. Inhibit the differentiation of 3T3-L1 preadipocyte cell line [55]. **In vivo:** 1. Inhibit obesity development in HFD-fed mice [55]. 2. Improving metabolic disorders like hyperlipidemia and hyperglycemia [55].	
Kirenol	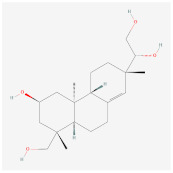	Wnt signaling pathway	**In vitro:** 1. Inhibit the differentiation of 3T3-L1 preadipocyte cell line [57].	
13m	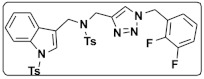	Wnt signaling pathway	**In vitro:** 1. Inhibit adipogenesis in 3T3-L1 and C3H10T1/2 cell line [56].	
C59	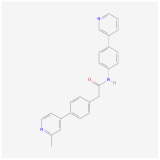	Wnt signaling pathway	**In vitro:** 1. Promote brown adipogenesis in stromal vascular fraction (SVF) from mouse inguinal white, most likely targeting beige precursor cells [58,59].	
XAV939	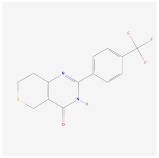	Wnt signaling pathway		
Hedgehog activator smoothened agonist (SAG)	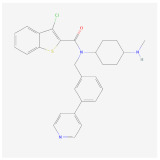	Hedgehog signaling pathway	**In vivo:** 1. Block white but not brown adipocyte differentiation in mice [71].	
Bimagrumab		BMP signaling pathway	**In vivo:** 1. Lead to significant loss of total body fat mass and metabolic improvement in overweight patients with type 2 diabetes [64].	NCT03005288, completed

## 4. Epigenetic Modification Compounds Affecting Adipogenesis

**Figure 2:**
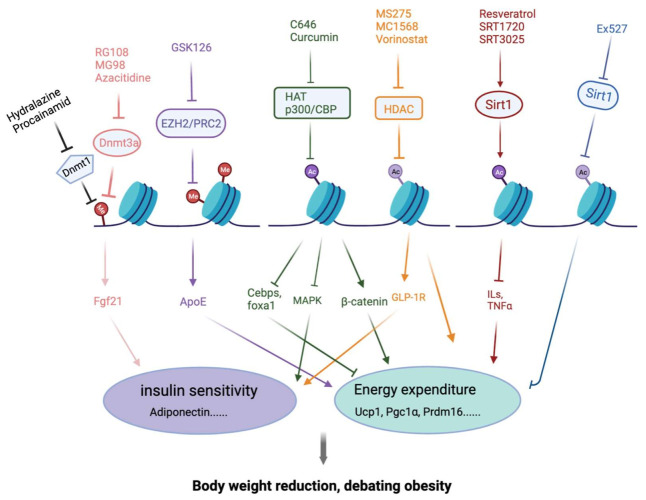
Compounds regulating adipogenesis through epigenetic modification. This figure describes the key components of the epigenetic machinery and its regulation by selected epigenetic compounds. Cellular and molecular mechanisms by which epigenetic compounds might mediate the prevention of obesity through regulating adipogenesis and eventually have an influence on insulin sensitivity and energy homeostasis. This figure summarizes epigenetic regulation in debating obesity, and their potential regulation by epigenetic compounds. Almost all compounds will affect energy expenditure. Abbreviations: DNMT, DNA methyl transferase; PRC2, poly-comb repressive complex 2; HAT, histone acetyltransferase; HDAC, histone deacetylase; HDM, histone demethylase; ILs, interleukins.

Epigenetic regulation is the basis for the genome-wide transcriptional programming that underlies many developmental processes, which refer to the regulation of gene expression without changing DNA sequences [84,85]. Wrapping with the octamer of histone H2A, H2B, H3 and H4, DNA molecules are packed into chromatin form. Epigenetic signals and chromatin-modifying proteins contribute to adipogenesis and phenotypic maintenance of the mature adipocytes. In this part, we intend to summarize the recently described epigenetic processes, natural products and compounds affecting adipogenesis and their connections with the major adipogenesis machinery and signal regulators. We focus on histone acetylation/deacetylation, histone methylation/demethylation, DNA methylation/demethylation and their intricate interplay (Figure 2, Table 2).

### 4.1. Histone Acetylation Modulators

Histone acetylation installs the covalent addition of acetyl groups to the lysine residues on histone H3 and H4 and to a lesser extent on H2A and H2B, which leads to looser binding between DNA and histone, and hence a relatively open structure. Generally, histone acetylation is associated with transcriptional activation. There are three subfamilies of histone acetyltransferases (HATs): GNAT, MYST and p300/CBP. HAT catalyzes the transfer of acetyl to the ε-amino-group of lysine from acetyl-CoA. In yeast Saccharomyces Cerevisiae, reduced expression of ACC1 results in increased histone acetylation globally [86]. As the cellular level of acetyl-CoA reflects overall energy metabolism, histone acetylation is modulated and coupled with energy metabolism [87]. Early studies using mouse fibroblast NIH-3T3 cell showed that CBP and p300 are involved in the different time points of in vitro adipogenesis for the induction of PPARγ [88]. CBP deficient mice showed a decreased weight in WAT, but not in any other tissues [89]. In addition, histone acetyltransferase GCN5 and PCAF redundantly regulated the brown adipogenesis in vitro through H3K9 acetylation, and activation of the expression of PPARγ and PRDM16. *Gcn5* and *Pcaf* double knockout in mouse brown fat significantly impaired the BAT development and function. Therefore, PPARγ induction is a critical link between histone acetylation with an energy-rich condition and adipogenesis.

As a known HAT inhibitor (HATi), Curcumin, a component of the spice turmeric, has been used in traditional Chinese medicine, as it is a relatively safe and inexpensive drug that has an effect on diabetes and liver disorders [90]. Curcumin also exhibits activity against obesity through multiple mechanisms, including regulating adipogenesis, and antioxidant and anti-inflammation activity. In adipogenesis specifically, curcumin treatment suppresses 3T3-L1 adipocyte differentiation, although there may be multiple mechanisms for this phenomenon. For example, Ahn et al. demonstrated that curcumin inhibits the activities of mitogen-activated protein kinases (MAPKs). Meanwhile, curcumin also restored β-catenin expression and activated Wnt signaling [91,92,93]. These mechanisms may function together with HAT inhibition and PPARγ induction. Furthermore, Curcumin showed beneficial effects on body weight reduction and energy metabolism in vivo. Two weeks of high dietary curcumin supplementation feeding in rats reduced epididymal adipose tissue and increased fatty acid β-oxidation, indicating an increase of energy expenditure after curcumin treatment [94,95,96]. In addition, dietary curcumin significantly improves obesity-associated inflammation and diabetes in mouse models of diabesity [96]. Furthermore, curcumin could stimulate human adipocyte differentiation in a dose-dependent manner, which can be mimicked by PPARγ agonist troglizatone [97,98].

It should be noted that curcumin also works systemically, in addition to adipogenesis. There were multiple reports showing that it suppresses the proinflammatory transcription factors and signaling, downregulates inflammatory cytokines and upregulates adiponectin and other gene products through working in macrophages, hepatocytes and other metabolism-related cells. These curcumin-induced alterations are helpful to reverse insulin resistance, hyperglycemia, hyperlipidemia, and other symptoms linked to obesity. Other structurally homologous nutraceuticals, derived from red chili, cinnamon, cloves, black pepper, and ginger, also exhibit effects against obesity and insulin resistance [99]. There are numerous studies using curcumin on obese subjects. In one clinical study, obese subjects were treated with a commercial formulation of curcumin supplemented with a bioavailability enhancer, piperine, for one month. Although there were no changes in weight, BMI or body fat, serum triglyceride levels were significantly decreased after curcumin treatment, indicating the improvement of insulin’s action [100].

Recently, there have been updated reports on the regulation of adipogenesis and metabolism by some more specific HAT inhibitors. One example is C646, a specific SMW inhibitor of CBP/p300 [101]. Nishimura et al. found that C646 treatment significantly decreased the adiposity in larval zebrafish [102]. More recently, it was shown that C646 blocked the proliferation, arrested the cell cycle and promoted the differentiation of goat adipose-derived stem cells (gADSCs) to adipocytes [103]. Overall, the effects of HATi on obesity need to be investigated. Genetic evidence supports further pharmacological study. The emerging of subtype selective HAT inhibitors, including C646 and A-485 [104], provides good tools for further in vitro and in vivo studies.

### 4.2. Histone Deacetylation Inhibitors

In contrast to HAT, histone deacetylases (HDACs) remove acetyl groups from histone, compacting the chromatin structure and preventing the binding of transcription factors. There are four classes of HDACs. Class I consists of HDAC1, 2, 3, and 8. Class II consists of HDAC4, 5, 6, 7, 9, and 10. Class III includes sirtuins 1–7, a unique group of nicotinamide adenine dinucleotide (NAD)-dependent enzymes and we will focus on this family in the next section. Class IV only consists of HDAC11 [105,106]. There are numerous studies showing that down-regulation or inhibition of class I HDACs stimulates adipocyte differentiation. The HDAC1 level was higher in white adipocytes than brown adipocytes, and deleting *Hdac1* promoted browning [107] and overcame the reduced in vitro adipogenesis by low intensity pulsed ultrasound in rat visceral preadipocytes. Indeed, as HDACs have long been recognized as an important target class for cancer treatment, many HDAC inhibitors with variable selectivity and potency have been developed and provide useful tools for the study of regulation in adipogenesis and metabolism.

Suberoylanilide hydroxamic acid (SAHA, vorinostat, Zolinza^®^), is a first-generation HDAC inhibitor used widely for the treatment of cancer in clinical practice [108]. When applied in cell culture, SAHA induced the uncommitted adipose-derived MSCs to differentiate [108]. SAHA treatment led to less lipid accumulation and smaller lipid droplets in cultured adipocytes. In vivo, SAHA reduced body weight gain and increased the core temperature in lean and obese mice through *Ucp1* upregulation. Through transcriptome-sequencing, a group of zinc finger proteins (Zfps) was found up-regulated by SAHA treatment, among which the knockdown of Zfp691 or Zfp719 largely abolishes SAHA-induced UCP1 expression in adipocytes. Therefore, SAHA stimulated histone H3 acetylation at Zfp719 promoter and enhanced the expression of it, which may further strengthen the UCP1 upregulation and energy expenditure. This mechanism is useful for treating obesity and related metabolic dysfunctions [109].

MS-275 (Entinostat) is a second-generation class I HDAC inhibitor, which is currently in clinical trials to treat solid tumors such as breast tumors and non-small cell lung tumors. It enhances the glucagon-like peptide-1 receptor (GLP-1R) agonism to potentiate glucose-stimulated insulin secretion and decreases body weight in DIO mice. The combination therapy of MS-275 and liraglutide caused a reduction of body weight gain and improved fasting glycemia in DIO mice [110]. MS-275 treated mice showed a 10% reduction of body weight, smaller adipocyte size, and improved glucose tolerance. MS-275 also promoted the browning of visceral and subcutaneous WAT by upregulation of PGC1a and UCP1 expression, although its effect in BAT was limited in term of UCP1 upregulation and reduction of brown adipocyte size [111]. The repression of Ucp1 by HDACs is likely only to function in WAT, where the effect of HDAC inhibitor on derepressing Ucp1 gene is most prominent. On the contrary, MC1568 is a selective class II HDAC inhibitor, which showed negative effects on brown fat thermogenesis. MC1568 upregulated Rb protein, leading to downregulation of Ucp1 and other functional markers [105]. In addition, MC1568 attenuates the RAR- and PPARγ-mediated adipogenesis [112]. These results suggest that HDAC subclass selective inhibitors may provide useful starting points to dissect their functions and efficacy in adipogenesis. Based on current evidence, Class I HDAC inhibitors may be worthy of further investigation. There are still multiple members in the class I HDAC including HDAC1, 2, 3 and 8. Chemical tools with higher selectivity toward individual class members would bring a better safety profile and have a better chance of further validation studies in the future.

### 4.3. Sirtuin Activators

The Sirtuins family of NAD^+^-dependent deacetylases (class III HDAC) includes seven members in mammals, SIRT1-7, which differ greatly in function and sub-cellular location. SIRT1 and SIRT2 are located in the nucleus and cytoplasm, while SIRT3-5 are mainly localized in mitochondria. A few of the SIRT family of enzymes have been investigated for their roles in adipogenesis [113,114]. SIRT1 is a negative regulator of adipogenesis. Reduction of SIRT1 resulted in increased expression of *Pparγ*, *Cebpα*, and *aP2*. Knockout of *Sirt1* promoted adipogenesis in vivo as well as decreasing the free fatty acid release from WAT [115,116]. Interestingly, an antagonism has been reported between SIRT7 and SIRT1. SIRT7 interacts with SIRT1 and inhibits its auto-deacetylation. In *Sirt7*-/- mice, SIRT1 level was high and there was a reduction in WAT as well as a decrease in expression of *Pparγ*, and *aP2* [113]. Small molecules that can modulate the activity of Sirtuins have been shown to have the potential for treating many human diseases such as T2D, cancer, rheumatoid arthritis, CVD and other age-related diseases.

Resveratrol (RSV) is a widely studied natural polyphenolic compound with potential pharmacological impact on cancer, CVD, obesity, aging, and T2D. In a randomized double-blind cross-over study, Timmers et al. showed that 150 mg/day of RSV treatment improved metabolic profile. RSV supplementation reduces adipocyte size in rhesus monkeys fed a high-fat, high sugar diet for 2 years [117]. Consistently, Konings et al. found that 30 days of RSV treatment (150 mg/day) decreased the size of abdominal subcutaneous adipocytes in obese men [118]. A meta-analysis of randomized and controlled trials shows that RSV supplement could significantly reduce body weight and fat mass without affecting leptin or adiponectin levels. Obesity is often linked to insulin resistance [119]. RSV supplementation was studied in patients with obesity and/or metabolic syndrome. Some reported an improvement in insulin sensitivity in response to RSV [120], while others failed to reach a similar conclusion [121]. Differences in protocol design and sample size may have contributed to these discrepancies. Obesity is often associated with elevated levels of inflammatory markers that promote vascular dysfunction [122]. Fat depots in obese individuals represent a major source of ROS that are released into the peripheral blood to affect many tissues and organs [123]. RSV also decreases diet-induced NF-κB activation and the steady-state mRNA levels of several inflammatory markers, such as IL-6 and IL-1β. Six-to twelve-month administration of RSV (350 mg per day) mediates the decrease in the production of IL-6 [124], IL-6/IL-10, and TNF-α [125] in patients with high cardiovascular risk.

Besides anti-inflammation and antioxidant, many of RSV’s effects are mediated via SIRT1 and AMPK activation [126,127]. Using it as a SIRT1 activator, Marie et al. found that PGC1α activity was induced by RSV treatment through SIRT1 activation, and RSV-treated mice showed improved insulin sensitivity [128]. SIRT1 over-expression in C3H10T1/2 increased PGC1α and PRDM16 protein level. Pharmacologic activation of SIRT1 by a more potent agonist SRT3025 increased *Foxc2*, *Pgc1α*, *Dio2*, *Tfam* and *Cyc1* expression, while inhibition of SIRT1 by EX527 down-regulated UCP1 in C3HT101/2 cells [129]. Besides these target genes, RSV treatment led to PPARγ activation and prevention of triglyceride accumulation [127,130,131]. As SIRT1 has been shown to directly deacetylate PPARγ in WAT, resveratrol would stimulate the browning of WAT, increase oxygen consumption and promote energy dissipation in the form of heat through this mechanism [132,133,134,135,136]. Thus, body weight may be reduced. Further studies in this direction are needed, especially when there is a more specific SIRT1 agonist.

### 4.4. Histone Methylation Modulators

Histones can be methylated in a variety of ways on the lysine and arginine residues. These modifications are usually catalyzed by site-specific histone methyltransferases (HMTs). The demethylation refers to the removal of the methyl groups, which is carried out by histone demethylase proteins (HDMs). Histone methylation can be correlated with either gene activation or repression, depending on the methylation sites. For example, methylation of histone H3 on lysine 4 (H3K4), lysine 36 (H3K36), or lysine 79 (H3K79) is correlated with gene activation, but methylation on lysine 9 (H3K9) or lysine 27 (H3K27) is correlated with gene repression [137]. SMW inhibitors targeting many HMTs or HDMs have been developed to treat multiple cancers and amnesia, and a few of these are in clinical trial studies. Some have also been tested in adipogenesis studies and showed interesting effects. Below are two examples.

Enhancer of Zeste Homolog 2 (EZH2) is a histone methyltransferase that plays a key role in cell stemness and differentiation by catalyzing trimethylation of histone H3K27 using S-adenosyl-methionine (SAM) as the methyl donor. It is always incorporated in the poly-comb repressive complex 2 (PRC2), together with SUZ12 and EED proteins, to efficiently catalyze the methylation reaction. GSK126 is a potent, highly selective, SAM-competitive, small-molecule inhibitor of EZH2 methyltransferase activity. GSK126 inhibited the differentiation of white adipocytes in vitro. GSK126 treatment in mice has been associated with reduced body fat and improved cold tolerance by promoting the differentiation of thermogenic beige adipocytes [138]. The GSK126-treated mice also exhibited improved glucose tolerance and increased lipolysis. Consistently, genetic deletion of EZH2 inhibited white adipocytes but promoted brown and beige adipocyte differentiation in mice. GSK126 could inhibit the differentiation from MEFs to white adipocytes but promote the differentiation from MEFs to brown/beige adipocytes [139]. Another study showed that GSK126 promoted lipoprotein-dependent lipid accumulation via inducing ApoE expression in adipocytes [140]. Together, EZH2/PRC2 modulates adipocyte differentiation and function, and further studies are warranted.

The second example is LSD1, a HDM reducing levels of histone methylation in both H3K4 and H3K9. There is much supporting evidence connecting it with adipogenesis, adipocyte maintenance and energy metabolism. Tissue-specific knockout of *Lsd1* in BAT induced brown-to-white adipocyte conversion through transcriptional factor NRF1 and CoREST complex [141]. Mechanistically, LSD1 could cooperate with ZFP516 to activate BAT-specific genes and to promote thermogenesis by demethylating H3K9 at the promoter of *Ucp1* [142]. Through epigenetic compound screening, Chen et al. identified LSD1 inhibitors as brown adipogenesis modulators. LSD1 could repress Wnt signaling by demethylating H3K4 on the promoter of multiple Wnt pathway genes, thereby promoting brown fat differentiation [143]. Overall, LSD1 plays an important role in modulating adipogenesis and regulating energy homeostasis.

### 4.5. DNA Methylation Modulators

DNA methylation is an inheritable epigenetic change, describing the adding of a methyl group to the C-5 position of the cytosine ring by DNA methyltransferases (DNMTs), usually in the context of a cytosine-guanine dinucleotide (CpG) doublet [144]. There are multiple members in the DNMT family including DNMT1, -3a, -3b, and -3L in mammals, of which the most abundant is DNMT1 [145]. Recent evidence suggested that DNA methylation undergoes dynamic and reversible changes in the cell differentiation process [144]. DNMT1 is involved in clonal expansion and the early stages of adipogenesis in vitro. High levels of DNMT1 expression are observed in the first 24 h following adipogenic induction, with a subsequent reduction. DNMT1 appears to promote the early stages of differentiation through the methylation of *Pparγ* and *Glut4* loci, thereby inhibiting their early expression [137]. Reversely, the DNA demethylase TET family includes three enzymes TET1-3, oxidizing 5 mC to 5 hydroxy-methyl-cytosines (5 hmC). Intriguingly, 5 hmC colocalizes with PPARγ protein at the target gene enhancers in 3T3-L1 adipocytes [146]. In addition, it was reported that TET1 could promote RXRα expression and adipogenesis through DNA demethylation [147].

Hydralazine is a direct-acting smooth muscle relaxant used for the treatment of hypertension. However, it was not until recently that the precise molecular targeting of hydralazine towards the DNMT1 was uncovered [148]. Hydralazine has also been noted to decrease body fat in animals and humans, increasing lipolysis in abdominal subcutaneous adipose tissue [149]. There are clinical trials investigating the efficacy of hydralazine in the treatment of diabetes. Procainamide is a partially competitive inhibitor of DNMT1 and does not act on other members of the DNMT family [150]. It reduces the affinity of DNMT1 to DNA and SAM. Derivatives of procainamide are also under optimization and evaluation for the more selective inhibition of DNMT1 [151]. Azacitidine (i.e., 5-Azacytidine) is a widely-used DNMT inhibitor with weak/no selectivity on DNMT1, DNMT3a or DNMT3b. Early studies found that it induced adipogenic differentiation of the MSC cell line C3H10T1/2. Besides nucleoside analogs, other SMW inhibitors of DNMT1 include MG98 and RG108. RG108 and Azacitidine treatment can relief the DNMT3a-mediated insulin resistance through *Fgf21* upregulation [152]. These compounds may serve as promising cancer therapeutics, as 5-Azacytidine is approved and in clinical use. It requires more in vivo and in human studies for validation in the treatment of obesity and other metabolic diseases.

**Table 2:** Compounds modulating the epigenetic regulation of adipogenesis.

Compound	Structure	Target	Experimental Conditions	NCT Numbers
Curcumin	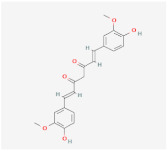	HAT	**In vitro:** 1. induction of PPARγ [97,98] 2. anti-inflammation and antioxidant [99] 3. inhibits MAPK, activates the β-catenin and Wnt signaling [91,92,93] **In vivo:** 1. reduced epididymal adipose tissue and increased fatty acid β-oxidation [96] **Clinical trials:** 1. decreased serum triglyceride levels [100]	1. NCT04723849, completed 2. NCT03864783, completed 3. NCT01975363, completed 4. NCT04315350, recruiting 5. NCT03542240, completed 6. NCT04595006, recruiting
C646	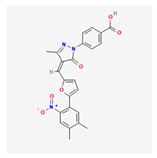	p300/CBP	**In vitro:** 1. increase TIP60 and PCAF expression, promoting adipocyte diffentiation [103] 2. decrease CEBPB, CEBPD, FOXA1, and FOXA2, having an influence on energy expenditure [102]	
MS-275	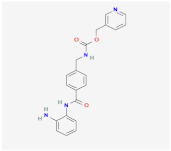	HDAC1–3	**In vitro:** 1. enhances GLP-1R agonism to potentiate insulin secretion [110] 2. upregulation of Pgc1a and UCP1 expression [111] **In vivo:** 1. decreases body weight in DIO mice [110]	
MC1568	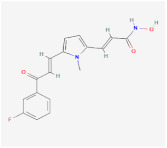	HDAC4,5,7 and 9	**In vitro:** 1. downregulation of Ucp1 [105] 2. attenuates the RAR- and PPARγ-mediated adipogenesis [112]	
Vorinostat	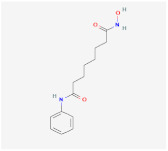	HDAC	**In vitro:** 1. less lipid accumulation and smaller lipid droplets [109] **In vivo:** 1. reduced body weight gain and increases the core temperature in lean and obese mice through Ucp1 upregulation [109]	
Resveratrol	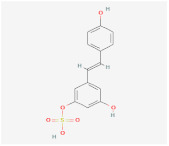	SIRT1 activator	**In vitro:** 1. upregulation of Pgc1a, prdm16 [128] **In vivo:** 1. reduce adipocyte size in rhesus monkeys fed a high-fat [123] **Clinical trials:** 1. protection against obesity-related comorbid conditions [123] 2. decreased the size of abdominal subcutaneous adipocytes [117] 3. decrease in the production of IL-6 , IL-6/IL-10, and TNF-α in patients with high cardiovascular risk [124,125] 4. reduce body weight and fat mass [118] 5. improve insulin sensitivity [119]	1. NCT02247596, completed 2. NCT04723849, completed 3. NCT01446276, completed 4. NCT01150955, completed 5. NCT01412645, completed 6. NCT00998504, completed 7. NCT01714102, completed 8. NCT01717820, completed 9. NCT02419092, Completed 10. NCT00823381, completed 11. NCT01302639, completed 12. NCT02216552, completed 13. NCT02381145, completed 14. NCT02633150, completed 15. NCT03448094, completed 16. NCT01518764, completed 17. NCT02114892, completed 18. NCT02767869, completed
SRT1720	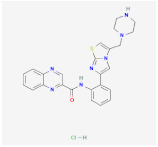	SIRT1 activator		
**SRT3025**	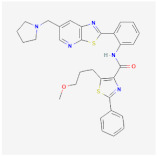		**In vitro:** 1. increased Foxc2, Pgc1α, Dio2, Tfam, and Cyc1 expression [129]	
Ex-527	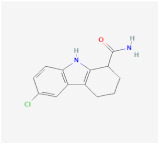	SIRT1 inhibitor	**In vitro:** 1. down-regulated UCP1 in C3HT101/2 cells [129]	
GSK126	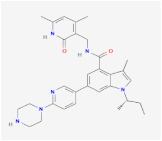	EZH2/PRC2	**In vitro:** 1. promote lipid accumulation via inducing ApoE expression [140]	
Hydralazine	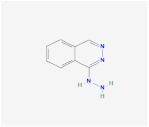	DNMT1	**In vivo:** 1. decrease body fat in animals and humans, increase lipolysis in abdominal subcutaneous adipose tissue [149]	
Procainamid	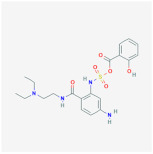	DNMT1	It reduces the affinity of DNMT1 to bind DNA and SAM	
RG108	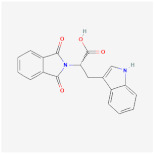	DNMT1	**In vitro:** 1. rescue Dnmt3a-mediated insulin resistance through Fgf21 upregulation with azacytidine [150]	
**Azacitidine**	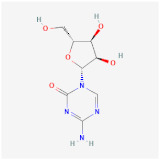	DNMT1	**In vitro:** 1. rescue Dnmt3a-mediated insulin resistance through Fgf21 upregulation with RG108 [150]	

## 5. Concluding Remarks and Perspectives

To date, basic and clinical research has enormously progressed our understanding of obesity as a chronic disease, with multiple organs involved and under the influence of both genetic and environmental factors. However, there is still an unmet need for effective anti-obesity therapeutics. Most currently available drugs still function by restricting energy intake, such as GLP1R agonists [11]. Here we reviewed multiple compounds regulating signaling or epigenetic status in adipogenesis. They provide material for research and may regulate other aspects of energy metabolism or obesity through variable mechanisms.

Many signaling cascades including AMPK, Wnt, BMP, Insulin or Hedgehog could influence the adipogenesis process and marker genes, such as *Cebpα*, *Pparγ* and *Cebpβ*. Wnt and Hedgehog signaling pathways play fundamental roles in multiple functions, especially development and adult stem cell maintenance, but manipulating these pathways may incur safety risk. The beneficial effects of AMPK activation on obesity and related metabolic conditions are well documented, and they may require activation of AMPK not only in adipose tissue but also liver, macrophage and muscle. However, systemic activation of AMPK led to heart hypertrophy. To circumvent this problem, one approach is to develop a tissue-specific AMPK agonist. Adipose-specific deletion of AMPK exhibits beneficial effects and supports this approach. Another approach is to identify the critical downstream player of AMPK in metabolism modulation and directly target that downstream player. This method would compensate usefully. In addition, bimagrumab, targeting the BMP receptor protein ActRII, may be of interest for further mechanistic and functional investigation. Epigenetic mechanisms modulating adipogenesis have been extensively studied using genetic-manipulated animal models. However, pharmacologic inhibitors are just emerging. The SIRT1 agonist resveratrol is a representative compound due to its application in clinical trials. Histone methylation and DNA methylation have been related to adipogenesis in research. Further steps in the clinic may be difficult and require careful study, as rodents and humans are different in many aspects. Research is proceeding on several fronts, but as with all potential drugs the common pitfalls of inadequate bioavailability, impractical pharmacokinetic or pharmacodynamic characteristics and adverse effects must be overcome.

Resveratrol and curcumin are natural products that modulate multiple signaling pathways or targets in chronic diseases, yet they are pharmacologically safe. Curcumin and resveratrol have potential in the prevention of chronic diseases such as dysregulated inflammation. Enhanced bioavailability of curcumin and resveratrol is likely to bring this promising natural product to the forefront of therapeutic agents for the treatment of human disease. Because most of the therapeutic effects of resveratrol and curcumin are based on cell culture and animal studies, more clinical trials are needed to fully realize their potential. Given that our knowledge of epigenetic modifications in diseases is incomplete, much needs to be accomplished to validate their potential in therapy.

Very recently, the results from single-cell RNA-sequencing revealed the possible lineage plasticity and heterogeneity of adipogenic precursors and shed new light on the markers and precursor subpopulations for individual fat depots [6]. A few very recent studies in epigenetic regulation of adipogenesis and thermogenesis revealed a possible common theme: different epigenetic regulators may work together and form networks to regulate the expression of the critical adipogenic factors. For example, the DNA demethylase TET1 coordinates with HDAC1 to regulate the expression of *Ucp1* and *Ppargc1a* in beige adipocytes [153]. Histone H3K27 demethylase UTX interacts with DNMT1 to regulate the diet-induced myogenesis in mouse BAT [154]. These new research landscapes will bring valuable insights into potential directions for the treatment of obesity.
